# *cis*-Chlorido(methyl­amine)­bis­(propane-1,3-diamine)­cobalt(III) dichloride monohydrate

**DOI:** 10.1107/S1600536813006442

**Published:** 2013-03-13

**Authors:** Velusamy Maheshwaran, Munisamy Manjunathan, Krishnamoorthy Anbalagan, Viswanathan Thiruselvam, Mondikalipudur Nanjappagounder Ponnuswamy

**Affiliations:** a Centre of Advanced Study in Crystallography and Biophysics, University of Madras, Guindy Campus, Chennai 600 025, India; b Department of Chemistry, Pondicherry University, Pondicherry 605 014, India

## Abstract

In the title compound, [CoCl(CH_5_N)(C_3_H_10_N_2_)_2_]Cl_2_·H_2_O, the Co^III^ ion has an octa­hedral coordination environment and is surrounded by four N atoms of two propane-1,3-diamine ligands in the equatorial plane, with another N atom of the methylamine ligand and a Cl atom occupying the axial positions. The crystal packing is stabilized by inter­molecular N—H⋯O, N—H⋯Cl, and O—H⋯Cl inter­actions, generating a three-dimensional network.

## Related literature

For the linear solvation energy relationship (LSER) method, see: Anbalagan (2011 ▶); Anbalagan *et al.* (2003 ▶, 2011 ▶). For the biological properties of cobalt(III) complexes, see: Chang *et al.* (2010 ▶). For related structures, see: Anbalagan *et al.* (2009 ▶); Lee *et al.* (2007 ▶); Ramesh *et al.* (2008 ▶); Ravichandran *et al.* (2009 ▶). For the preparation of (1,3-diamino­propane)­cobalt(III), see: Bailar & Work (1946 ▶).
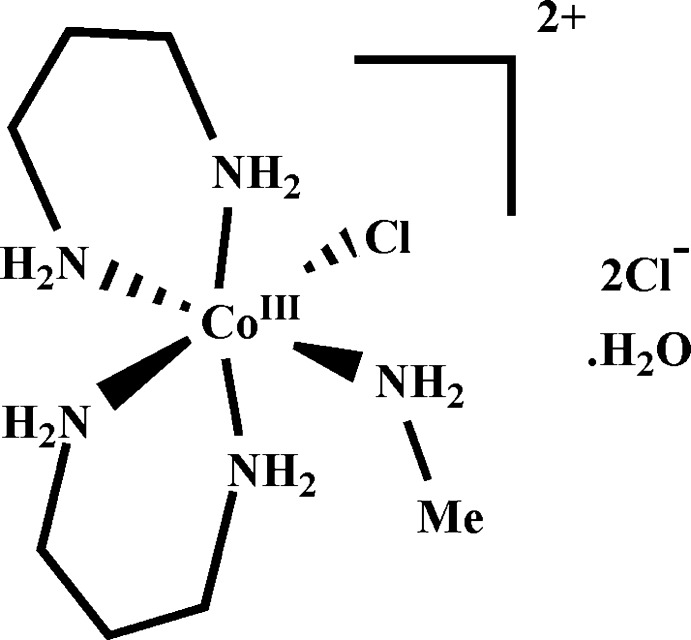


## Experimental

### 

#### Crystal data

[CoCl(CH_5_N)(C_3_H_10_N_2_)_2_]Cl_2_·H_2_O
*M* *_r_* = 362.62
Triclinic, 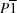

*a* = 7.4752 (6) Å
*b* = 7.9065 (6) Å
*c* = 14.4663 (13) Å
α = 76.022 (6)°
β = 76.907 (7)°
γ = 73.779 (4)°
*V* = 784.96 (11) Å^3^
*Z* = 2
Mo *K*α radiation
μ = 1.60 mm^−1^
*T* = 292 K
0.35 × 0.35 × 0.35 mm

#### Data collection

Oxford Diffraction Xcalibur Eos diffractometer
Absorption correction: multi-scan (*CrysAlis PRO*; Oxford Diffraction, 2009 ▶) *T*_min_ = 0.798, *T*_max_ = 1.000
5020 measured reflections
2764 independent reflections
2071 reflections with *I* > 2σ(*I*)
*R*_int_ = 0.027

#### Refinement

*R*[*F* ^2^ > 2σ(*F* ^2^)] = 0.030
*wR*(*F* ^2^) = 0.055
*S* = 0.92
2764 reflections
162 parameters
H atoms treated by a mixture of independent and constrained refinement
Δρ_max_ = 0.32 e Å^−3^
Δρ_min_ = −0.28 e Å^−3^

### 

Data collection: *CrysAlis CCD* (Oxford Diffraction, 2009 ▶); cell refinement: *CrysAlis RED* (Oxford Diffraction, 2009 ▶); data reduction: *CrysAlis RED*; program(s) used to solve structure: *SHELXS97* (Sheldrick, 2008 ▶); program(s) used to refine structure: *SHELXL97* (Sheldrick, 2008 ▶); molecular graphics: *ORTEP-3 for Windows* (Farrugia, 2012 ▶) and *PLATON* (Spek, 2009 ▶); software used to prepare material for publication: *PLATON*.

## Supplementary Material

Crystal structure: contains datablock(s) global, I. DOI: 10.1107/S1600536813006442/bt6894sup1.cif

Structure factors: contains datablock(s) I. DOI: 10.1107/S1600536813006442/bt6894Isup2.hkl

Additional supplementary materials:  crystallographic information; 3D view; checkCIF report

## Figures and Tables

**Table 1:** Hydrogen-bond geometry (Å, °)

*D*—H⋯*A*	*D*—H	H⋯*A*	*D*⋯*A*	*D*—H⋯*A*
N1—H1*C*⋯O1	0.90	2.12	2.960 (3)	155
N1—H1*D*⋯Cl2	0.90	2.43	3.317 (2)	170
N2—H2*C*⋯Cl3^i^	0.90	2.57	3.462 (2)	172
N2—H2*D*⋯Cl3	0.90	2.44	3.3196 (19)	164
N3—H3*C*⋯O1	0.90	2.04	2.880 (3)	155
N3—H3*D*⋯Cl3	0.90	2.50	3.3041 (18)	149
N4—H4*C*⋯Cl3^ii^	0.90	2.65	3.486 (2)	155
N4—H4*D*⋯Cl2	0.90	2.45	3.348 (2)	177
N5—H5*C*⋯Cl3^i^	0.90	2.49	3.359 (2)	162
N5—H5*D*⋯Cl3^ii^	0.90	2.37	3.2649 (19)	172
O1—H1*E*⋯Cl2^iii^	0.82 (4)	2.29 (4)	3.093 (3)	168 (4)
O1—H1*F*⋯Cl2^iv^	0.87 (4)	2.25 (4)	3.112 (3)	174 (3)

Symmetry codes: (i) 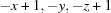; (ii) 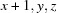; (iii) 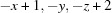; (iv) 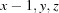.

**Fig. 1.**
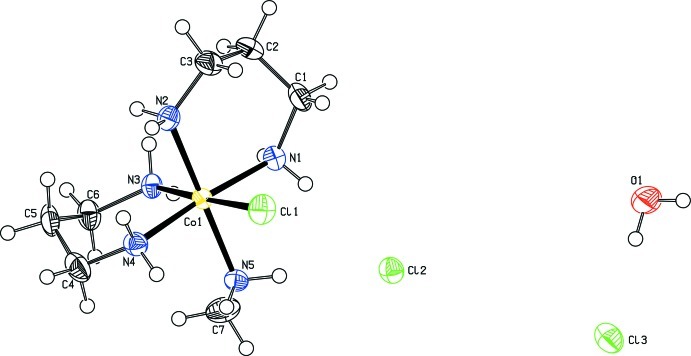
The molecular structure of the title compound, showing the atomic numbering and displacement ellipsoids drawn at 30% probability level.

**Fig. 2.**
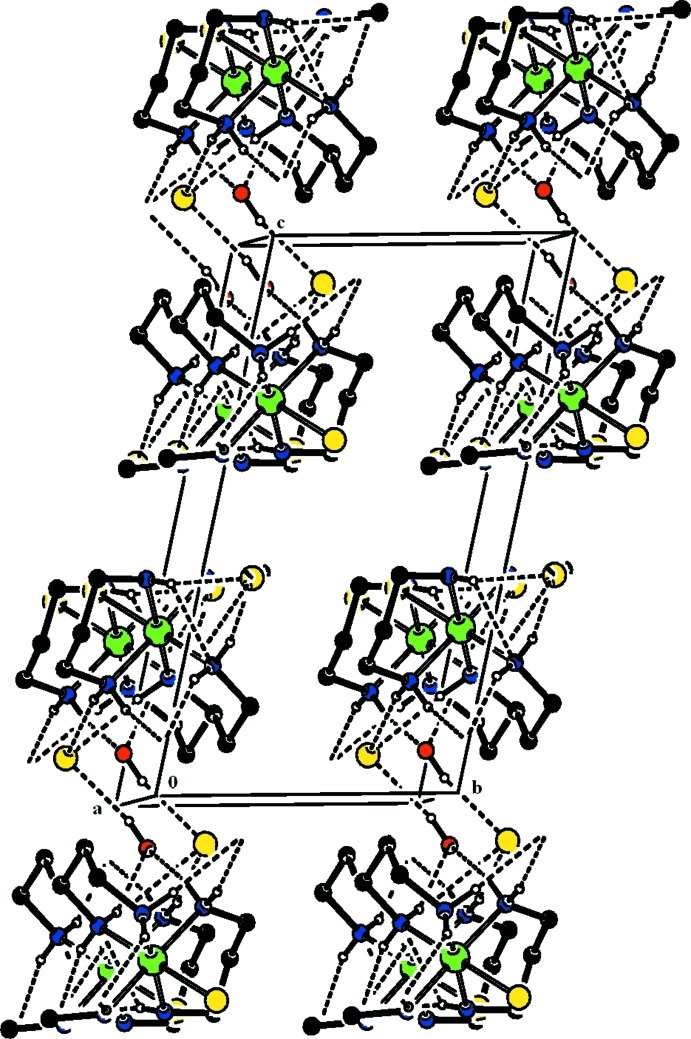
The packing of the molecules viewed down a axis.

## supplementary crystallographic information

### Comment

The interest in understanding the *outer-sphere electron-transfer* (OSET) reactions of transition metal complexes in mixed solvents has increased significantly in recent years. It was established that the method of *linear solvation energy relationship* (LSER) is a generalized treatment of solvation effects and can very well be used to understand the influence of solvent on reaction rates (Anbalagan *et al.*, 2003). The present research is the design and synthesis of cobalt(III) complexes with an objective to understand the structure-reactivity correlation. Substituting an amino ligand for the MeNH_2_ moiety can yield complexes of similar structure, but with differing electron transfer rate (Anbalagan, 2011; Anbalagan *et al.*, 2011).

Such complexes can offer a clear correlation between structure and spectral characteristics, reactions in particular. The optical properties and mechanism of electron transfer reaction can be understood through the structure of these complexes.

In addition cobalt(III) complexes have received a sustained high level of attention due to their relevance in various redox processes in biological systems and act as promising agents for antitumor, anthelmintic, antiparasitic, antibiotics and antimicrobial activities, as well as their multiple applications in fields such as medicine and drug delivery (Chang *et al.*,2010). Against this background and to ascertain the molecular structure and conformation, the X-ray crystal structure determination of the title compound has been carried out.

The *ORTEP* plot of the molecule is shown in Fig. 1. The molecular structure is symmetric with respect to cobalt, the Co^III^ ion has an octahedral coordination environment and is surrounded by four N atoms in an equatorial plane, with the other N and Cl atoms occupying the axial positions. The bond lengths [Co—N] and [Co—Cl] are comparable with the values reported in the literature (Lee *et al.*, 2007; Ramesh *et al.*, 2008; Anbalagan *et al.*, 2009; Ravichandran *et al.*, 2009).

The packing of the molecules viewed down *a* axis is shown in Fig. 2. The molecules are stabilized by N—H···Cl, N—H···O and O—H···Cl intermolecular interactions generating a three-dimensional network.

### Experimental

Two grams of *trans*-[CoIII(tn)_2_Cl_2_]Cl solid was made in to paste using 3–4 drops of water. To the solid mass, about 0.12*M* methyl amine (MeNH_2_) was added in drops for 20 min and mixed by grinding (Bailar & Work 1946). The grinding of the resulting dull green paste was continued to obtain red mass. The reaction mixture was set aside until no further change occurred and the product was allowed to stand overnight. Finally, the solid was washed and recrystallized using acidified water pre-heated to 70°C. The pure crystals were filtered, washed with ethanol and dried over vacuum. The microcrystalline solid obtained was pink colored and the yield was estimated to be 0.85 g (85%). X-ray quality crystals were grown after repeated recrystallization and using hot acidified water.

### Refinement

N and C-bound H atoms were positioned geometrically (N–H =0.90 Å, C–H =0.93–0.97 Å) and allowed to ride on their parent atoms, with *U*_iso_(H) =1.5*U*_eq_(C) for methyl H atoms and 1.2*U*_eq_(C,N) for all other H atoms. The water H atoms were freely refined.

### Crystal data

**Table:**

[CoCl(CH_5_N)(C_3_H_10_N_2_)_2_]Cl_2_·H_2_O	*Z* = 2
*M**_r_* = 362.62	*F*(000) = 380
Triclinic, *P*1	*D*_x_ = 1.534 Mg m^−^^3^
Hall symbol: -P 1	Mo *K*α radiation, λ = 0.71073 Å
*a* = 7.4752 (6) Å	Cell parameters from 2764 reflections
*b* = 7.9065 (6) Å	θ = 2.8–25.0°
*c* = 14.4663 (13) Å	µ = 1.60 mm^−^^1^
α = 76.022 (6)°	*T* = 292 K
β = 76.907 (7)°	Block, violet
γ = 73.779 (4)°	0.35 × 0.35 × 0.35 mm
*V* = 784.96 (11) Å^3^	

### Data collection

**Table:**

Oxford Diffraction Xcalibur Eos diffractometer	2764 independent reflections
Radiation source: Enhance(Mo)X-ray Source	2071 reflections with *I* > 2σ(*I*)
Graphite monochromator	*R*_int_ = 0.027
ω scans	θ_max_ = 25.0°, θ_min_ = 2.7°
Absorption correction: multi-scan (*CrysAlis PRO*; Oxford Diffraction, 2009)	*h* = −8→8
*T*_min_ = 0.798, *T*_max_ = 1.000	*k* = −6→9
5020 measured reflections	*l* = −17→16

### Refinement

**Table:**

Refinement on *F*^2^	Primary atom site location: structure-invariant direct methods
Least-squares matrix: full	Secondary atom site location: difference Fourier map
*R*[*F*^2^ > 2σ(*F*^2^)] = 0.030	Hydrogen site location: inferred from neighbouring sites
*wR*(*F*^2^) = 0.055	H atoms treated by a mixture of independent and constrained refinement
*S* = 0.92	*w* = 1/[σ^2^(*F*_o_^2^) + (0.0217*P*)^2^] where *P* = (*F*_o_^2^ + 2*F*_c_^2^)/3
2764 reflections	(Δ/σ)_max_ = 0.001
162 parameters	Δρ_max_ = 0.32 e Å^−^^3^
0 restraints	Δρ_min_ = −0.28 e Å^−^^3^

### Special details

**Table:**

Geometry. All e.s.d.'s (except the e.s.d. in the dihedral angle between two l.s. planes) are estimated using the full covariance matrix. The cell e.s.d.'s are taken into account individually in the estimation of e.s.d.'s in distances, angles and torsion angles; correlations between e.s.d.'s in cell parameters are only used when they are defined by crystal symmetry. An approximate (isotropic) treatment of cell e.s.d.'s is used for estimating e.s.d.'s involving l.s. planes.
Refinement. Refinement of *F*^2^ against ALL reflections. The weighted *R*-factor *wR* and goodness of fit *S* are based on *F*^2^, conventional *R*-factors *R* are based on *F*, with *F* set to zero for negative *F*^2^. The threshold expression of *F*^2^ > σ(*F*^2^) is used only for calculating *R*-factors(gt) *etc*. and is not relevant to the choice of reflections for refinement. *R*-factors based on *F*^2^ are statistically about twice as large as those based on *F*, and *R*- factors based on ALL data will be even larger.

### Fractional atomic coordinates and isotropic or equivalent isotropic displacement parameters (Å^2^)

**Table:**

	*x*	*y*	*z*	*U*_iso_*/*U*_eq_	
C1	0.3821 (4)	0.4046 (4)	0.7738 (2)	0.0358 (7)	
H1A	0.4513	0.4943	0.7385	0.043*	
H1B	0.3086	0.4444	0.8324	0.043*	
C2	0.2490 (4)	0.3907 (4)	0.7126 (2)	0.0367 (7)	
H2A	0.1912	0.2912	0.7441	0.044*	
H2B	0.1490	0.4997	0.7074	0.044*	
C3	0.3492 (4)	0.3631 (4)	0.61316 (19)	0.0346 (7)	
H3A	0.2561	0.3784	0.5729	0.042*	
H3B	0.4205	0.4540	0.5850	0.042*	
C4	0.8945 (4)	−0.1858 (4)	0.8474 (2)	0.0381 (7)	
H4A	0.9755	−0.1910	0.8922	0.046*	
H4B	0.9572	−0.2780	0.8092	0.046*	
C5	0.7081 (4)	−0.2246 (4)	0.90423 (19)	0.0368 (7)	
H5A	0.7331	−0.3273	0.9561	0.044*	
H5B	0.6347	−0.1223	0.9331	0.044*	
C6	0.5945 (4)	−0.2629 (4)	0.84015 (19)	0.0328 (7)	
H6A	0.6729	−0.3573	0.8064	0.039*	
H6B	0.4873	−0.3057	0.8804	0.039*	
C7	0.8086 (4)	−0.2353 (4)	0.5976 (2)	0.0394 (8)	
H7A	0.9010	−0.2769	0.5449	0.059*	
H7B	0.6842	−0.2259	0.5863	0.059*	
H7C	0.8299	−0.3188	0.6567	0.059*	
N1	0.5177 (3)	0.2334 (3)	0.80003 (14)	0.0256 (5)	
H1C	0.4534	0.1636	0.8464	0.031*	
H1D	0.6020	0.2573	0.8277	0.031*	
N2	0.4793 (3)	0.1835 (3)	0.61329 (15)	0.0243 (5)	
H2C	0.5452	0.1837	0.5529	0.029*	
H2D	0.4070	0.1045	0.6240	0.029*	
N3	0.5246 (3)	−0.1013 (3)	0.76779 (14)	0.0226 (5)	
H3C	0.4124	−0.0437	0.7971	0.027*	
H3D	0.4999	−0.1426	0.7204	0.027*	
N4	0.8692 (3)	−0.0082 (3)	0.78236 (15)	0.0273 (5)	
H4C	0.9792	−0.0075	0.7412	0.033*	
H4D	0.8549	0.0732	0.8191	0.033*	
N5	0.8253 (3)	−0.0572 (3)	0.60532 (14)	0.0251 (5)	
H5C	0.8120	0.0139	0.5473	0.030*	
H5D	0.9456	−0.0715	0.6121	0.030*	
O1	0.2225 (3)	0.0443 (4)	0.91094 (19)	0.0487 (6)	
Cl1	0.81500 (9)	0.30498 (10)	0.62727 (5)	0.03471 (19)	
Cl2	0.81288 (10)	0.28479 (10)	0.92550 (5)	0.0405 (2)	
Cl3	0.27376 (8)	−0.14018 (9)	0.61638 (5)	0.02962 (18)	
Co1	0.66519 (4)	0.08437 (5)	0.70286 (3)	0.01944 (10)	
H1E	0.208 (5)	−0.031 (5)	0.960 (3)	0.085 (16)*	
H1F	0.112 (5)	0.118 (5)	0.915 (2)	0.074 (13)*	

### Atomic displacement parameters (Å^2^)

**Table:**

	*U*^11^	*U*^22^	*U*^33^	*U*^12^	*U*^13^	*U*^23^
C1	0.0395 (17)	0.0269 (19)	0.038 (2)	0.0020 (14)	−0.0084 (14)	−0.0098 (15)
C2	0.0274 (15)	0.035 (2)	0.0400 (19)	0.0078 (13)	−0.0095 (13)	−0.0063 (15)
C3	0.0406 (17)	0.0266 (19)	0.0338 (19)	−0.0012 (14)	−0.0166 (14)	0.0013 (15)
C4	0.0400 (17)	0.036 (2)	0.039 (2)	−0.0008 (14)	−0.0218 (14)	−0.0033 (16)
C5	0.0546 (19)	0.030 (2)	0.0233 (17)	−0.0063 (15)	−0.0166 (14)	0.0051 (14)
C6	0.0447 (17)	0.0234 (18)	0.0310 (18)	−0.0128 (14)	−0.0097 (13)	0.0023 (14)
C7	0.0360 (16)	0.039 (2)	0.047 (2)	−0.0105 (15)	0.0020 (14)	−0.0226 (16)
N1	0.0262 (12)	0.0290 (15)	0.0244 (13)	−0.0090 (11)	−0.0051 (10)	−0.0071 (11)
N2	0.0257 (11)	0.0249 (15)	0.0224 (13)	−0.0057 (10)	−0.0058 (9)	−0.0036 (11)
N3	0.0265 (11)	0.0235 (14)	0.0193 (13)	−0.0077 (10)	−0.0049 (9)	−0.0040 (11)
N4	0.0233 (11)	0.0304 (16)	0.0291 (14)	−0.0042 (10)	−0.0070 (10)	−0.0077 (12)
N5	0.0210 (11)	0.0279 (15)	0.0254 (13)	−0.0042 (10)	−0.0043 (9)	−0.0047 (11)
O1	0.0358 (14)	0.0492 (18)	0.0457 (16)	−0.0074 (12)	0.0087 (11)	0.0023 (13)
Cl1	0.0364 (4)	0.0318 (5)	0.0377 (5)	−0.0194 (3)	−0.0022 (3)	−0.0007 (4)
Cl2	0.0464 (4)	0.0318 (5)	0.0444 (5)	−0.0038 (4)	−0.0175 (4)	−0.0069 (4)
Cl3	0.0260 (4)	0.0332 (5)	0.0315 (4)	−0.0087 (3)	−0.0089 (3)	−0.0039 (3)
Co1	0.01893 (18)	0.0188 (2)	0.0209 (2)	−0.00540 (15)	−0.00410 (14)	−0.00248 (16)

### Geometric parameters (Å, º)

**Table:**

C1—N1	1.471 (3)	C7—H7A	0.9600
C1—C2	1.514 (3)	C7—H7B	0.9600
C1—H1A	0.9700	C7—H7C	0.9600
C1—H1B	0.9700	N1—Co1	1.988 (2)
C2—C3	1.500 (4)	N1—H1C	0.9000
C2—H2A	0.9700	N1—H1D	0.9000
C2—H2B	0.9700	N2—Co1	1.9854 (19)
C3—N2	1.480 (3)	N2—H2C	0.9000
C3—H3A	0.9700	N2—H2D	0.9000
C3—H3B	0.9700	N3—Co1	1.9722 (18)
C4—N4	1.478 (3)	N3—H3C	0.9000
C4—C5	1.520 (4)	N3—H3D	0.9000
C4—H4A	0.9700	N4—Co1	1.987 (2)
C4—H4B	0.9700	N4—H4C	0.9000
C5—C6	1.519 (3)	N4—H4D	0.9000
C5—H5A	0.9700	N5—Co1	1.9815 (19)
C5—H5B	0.9700	N5—H5C	0.9000
C6—N3	1.493 (3)	N5—H5D	0.9000
C6—H6A	0.9700	O1—H1E	0.82 (4)
C6—H6B	0.9700	O1—H1F	0.87 (4)
C7—N5	1.480 (3)	Cl1—Co1	2.2599 (7)
			
N1—C1—C2	112.7 (2)	Co1—N1—H1C	106.8
N1—C1—H1A	109.0	C1—N1—H1D	106.8
C2—C1—H1A	109.0	Co1—N1—H1D	106.8
N1—C1—H1B	109.0	H1C—N1—H1D	106.7
C2—C1—H1B	109.0	C3—N2—Co1	121.69 (15)
H1A—C1—H1B	107.8	C3—N2—H2C	106.9
C3—C2—C1	111.9 (2)	Co1—N2—H2C	106.9
C3—C2—H2A	109.2	C3—N2—H2D	106.9
C1—C2—H2A	109.2	Co1—N2—H2D	106.9
C3—C2—H2B	109.2	H2C—N2—H2D	106.7
C1—C2—H2B	109.2	C6—N3—Co1	124.59 (14)
H2A—C2—H2B	107.9	C6—N3—H3C	106.2
N2—C3—C2	112.7 (2)	Co1—N3—H3C	106.2
N2—C3—H3A	109.1	C6—N3—H3D	106.2
C2—C3—H3A	109.1	Co1—N3—H3D	106.2
N2—C3—H3B	109.1	H3C—N3—H3D	106.4
C2—C3—H3B	109.1	C4—N4—Co1	122.49 (15)
H3A—C3—H3B	107.8	C4—N4—H4C	106.7
N4—C4—C5	112.6 (2)	Co1—N4—H4C	106.7
N4—C4—H4A	109.1	C4—N4—H4D	106.7
C5—C4—H4A	109.1	Co1—N4—H4D	106.7
N4—C4—H4B	109.1	H4C—N4—H4D	106.6
C5—C4—H4B	109.1	C7—N5—Co1	124.77 (16)
H4A—C4—H4B	107.8	C7—N5—H5C	106.1
C6—C5—C4	111.5 (2)	Co1—N5—H5C	106.1
C6—C5—H5A	109.3	C7—N5—H5D	106.1
C4—C5—H5A	109.3	Co1—N5—H5D	106.1
C6—C5—H5B	109.3	H5C—N5—H5D	106.3
C4—C5—H5B	109.3	H1E—O1—H1F	102 (3)
H5A—C5—H5B	108.0	N3—Co1—N5	93.88 (8)
N3—C6—C5	112.6 (2)	N3—Co1—N2	88.79 (8)
N3—C6—H6A	109.1	N5—Co1—N2	87.65 (8)
C5—C6—H6A	109.1	N3—Co1—N4	95.58 (8)
N3—C6—H6B	109.1	N5—Co1—N4	89.08 (9)
C5—C6—H6B	109.1	N2—Co1—N4	174.72 (8)
H6A—C6—H6B	107.8	N3—Co1—N1	89.21 (8)
N5—C7—H7A	109.5	N5—Co1—N1	176.46 (7)
N5—C7—H7B	109.5	N2—Co1—N1	94.16 (8)
H7A—C7—H7B	109.5	N4—Co1—N1	88.88 (8)
N5—C7—H7C	109.5	N3—Co1—Cl1	177.67 (7)
H7A—C7—H7C	109.5	N5—Co1—Cl1	87.31 (6)
H7B—C7—H7C	109.5	N2—Co1—Cl1	89.26 (6)
C1—N1—Co1	122.04 (16)	N4—Co1—Cl1	86.43 (6)
C1—N1—H1C	106.8	N1—Co1—Cl1	89.67 (6)
			
N1—C1—C2—C3	−69.3 (3)	C7—N5—Co1—N4	97.9 (2)
C1—C2—C3—N2	69.8 (3)	C7—N5—Co1—Cl1	−175.7 (2)
N4—C4—C5—C6	72.5 (3)	C3—N2—Co1—N3	113.74 (19)
C4—C5—C6—N3	−67.9 (3)	C3—N2—Co1—N5	−152.33 (19)
C2—C1—N1—Co1	48.1 (3)	C3—N2—Co1—N1	24.62 (19)
C2—C3—N2—Co1	−49.0 (3)	C3—N2—Co1—Cl1	−64.99 (18)
C5—C6—N3—Co1	35.5 (3)	C4—N4—Co1—N3	11.6 (2)
C5—C4—N4—Co1	−42.6 (3)	C4—N4—Co1—N5	−82.2 (2)
C6—N3—Co1—N5	81.2 (2)	C4—N4—Co1—N1	100.7 (2)
C6—N3—Co1—N2	168.8 (2)	C4—N4—Co1—Cl1	−169.5 (2)
C6—N3—Co1—N4	−8.2 (2)	C1—N1—Co1—N3	−113.12 (18)
C6—N3—Co1—N1	−97.0 (2)	C1—N1—Co1—N2	−24.39 (19)
C7—N5—Co1—N3	2.3 (2)	C1—N1—Co1—N4	151.28 (19)
C7—N5—Co1—N2	−86.3 (2)	C1—N1—Co1—Cl1	64.84 (17)

### Hydrogen-bond geometry (Å, º)

**Table:**

*D*—H···*A*	*D*—H	H···*A*	*D*···*A*	*D*—H···*A*
N1—H1*C*···O1	0.90	2.12	2.960 (3)	155
N1—H1*D*···Cl2	0.90	2.43	3.317 (2)	170
N2—H2*C*···Cl3^i^	0.90	2.57	3.462 (2)	172
N2—H2*D*···Cl3	0.90	2.44	3.3196 (19)	164
N3—H3*C*···O1	0.90	2.04	2.880 (3)	155
N3—H3*D*···Cl3	0.90	2.50	3.3041 (18)	149
N4—H4*C*···Cl3^ii^	0.90	2.65	3.486 (2)	155
N4—H4*D*···Cl2	0.90	2.45	3.348 (2)	177
N5—H5*C*···Cl3^i^	0.90	2.49	3.359 (2)	162
N5—H5*D*···Cl3^ii^	0.90	2.37	3.2649 (19)	172
O1—H1*E*···Cl2^iii^	0.82 (4)	2.29 (4)	3.093 (3)	168 (4)
O1—H1*F*···Cl2^iv^	0.87 (4)	2.25 (4)	3.112 (3)	174 (3)

Symmetry codes: (i) −*x*+1, −*y*, −*z*+1; (ii) *x*+1, *y*, *z*; (iii) −*x*+1, −*y*, −*z*+2; (iv) *x*−1, *y*, *z*.
